# Synoviocyte–chondrocyte triculture model for early-stage PTOA: fibronectin fragment-induced catabolic effects *in vitro* and *in vivo*

**DOI:** 10.3389/fbioe.2025.1683333

**Published:** 2025-12-04

**Authors:** Shahid Khan, Hongsik Cho, Karen A. Hasty, Taylor Brown, Sarayu Bhogoju, Anuradha Subramanian

**Affiliations:** 1 Department of Biotechnology Science and Engineering logical Sciences, University of Alabama-Huntsville, Huntsville, AL, United States; 2 Department of Orthopaedic Surgery and Biomedical Engineering, University of Tennessee Health Sciences Center-Campbell Clinic, Memphis, TN, United States; 3 VA Medical Center, Memphis, TN, United States; 4 Department of Chemical and Materials Engineering, University of Alabama-Huntsville, Huntsville, AL, United States

**Keywords:** post-traumatic osteoarthritis, post-traumatic osteoarthritis model, macrophages, fibroblasts-like synoviocytes, chondrocytes, THP-1 monocytes, fibronectin fragments, damage-associated molecular pattern

## Abstract

Post-traumatic osteoarthritis (PTOA), a subtype of osteoarthritis initiated by joint trauma, is driven by unresolved early inflammation that ultimately leads to cartilage degeneration. Although animal models have advanced our understanding of disease progression, they offer limited resolution of the early molecular events following trauma. In this study, we developed a transwell-based *in vitro* triculture model mimicking the early joint environment post-injury, incorporating macrophages, fibroblast-like synoviocytes (FLSs), and human articular chondrocytes (HACs). In lieu of the commonly used macrophage activator, lipopolysaccharide (LPS), this study utilizes fibronectin fragments (Fnfs), which belong to the damage-associated molecules released upon trauma to cartilage, to activate macrophages and simulate post-traumatic inflammation. The triculture was maintained for 12 days while promoting paracrine-only communication between the cell types. The activation of macrophages by Fnfs led to a sustained expression of pNFκB in both HACs and FLSs, as shown by immunofluorescence, alongside increased gene expression of inflammatory mediators MMP3, MMP13, and TNF-α. Fnfs triggered catabolic signaling across all joint-resident cell types used in this model. To support the translational relevance of the *in vitro* findings, a complementary *in vivo* experiment in which Fnfs were injected intra-articularly showed increased MMP activity gene expression and reduced COL2A1 gene expression in joint cartilage. The cytokine and gene expression profiles observed in the triculture model closely mirrored those observed in early-stage *in vivo* PTOA models and in the patient-derived synovial fluid obtained in the early traumatic phase, underscoring the model’s physiological relevance. This triculture platform captures the key aspects of early PTOA processes driven by macrophage activation and offers a biologically relevant tool for mechanistic studies and therapeutic screening.

## 1 Introduction

Post-traumatic osteoarthritis (PTOA), a subset of osteoarthritis (OA), results from maladaptive and incomplete repair mechanisms upon acute physical trauma or from changes that can result from chronic aberrant loading (Dilley et al., 2023). Use of analgesics, weight loss, and exercise can partially relieve symptoms and slow down the progression, but not halt it. Thus, the biomedical burden of PTOA and other cartilage pathologies is large, growing, and inadequately addressed. The reasons for this are multiple: 1) the exact onset of disease is difficult to identify, 2) disease progression is slow, and 3) the etiology and pathogenesis are incompletely understood; thus, there is no known cure (Dilley et al., 2023).

Recent studies suggest that the early inflammatory response contributes to PTOA and drives both the loss of tissue function and structural decline (Haseeb and Haqqi, 2013; Griffin and Scanzello, 2019). Briefly, a traumatic impact to the articular surface, with or without subchondral bone fracture and displacement, results in localized tissue damage, known as chondrocyte necrosis, initiating a cyclic cascade leading to the release of damage-associated molecular patterns (DAMPs), which consist of ECM degradation products such as catabolic fibronectin fragments (Fnfs), cartilage debris, and alarmins (Haseeb and Haqqi, 2013). Specifically, Fnfs robustly activate synovial macrophages via the stimulation of toll-like receptors (TLRs), leading to an increased expression of pro-inflammatory cytokines and inducing a strong temporal upregulation of catabolic markers (e.g., MMPs and ADAMTS) by synovial fibroblasts and chondrocytes (Peters et al., 2002; Blasioli and Kaplan, 2014; Evers et al., 2022). If unresolved, a cyclic cascade of inflammatory events leads to further and ongoing joint damage. New approaches are needed to understand and control early inflammatory processes in PTOA to protect joint tissues from inflammatory stress, thereby slowing down PTOA and OA progression and alleviating joint pain and disability.

Animal models for studying early PTOA have been developed and remain vital in testing the efficacy of small-molecule antioxidants, therapeutics, and metal-loaded nanoparticles (Cho et al., 2016; Bhatti et al., 2022). However, to delineate the inflammatory responses and separate complex molecular interactions in the early phase of PTOA, the use of animal models imposes certain limitations (Teeple et al., 2013). It is rationalized that *in vitro* models will be invaluable in ascertaining the mechanistic basis and providing a comprehensive map of the inflammatory processes in early PTOA.

As the molecular phenotypes for different types or stages of OA show distinct differences (e.g. RA vs. OA vs. early PTOA), the *in vitro* models being developed to represent a particular type of OA should consider the subtleties (Muenzebrock et al., 2022). Current methods that utilize monolayer cultures of primary cells exposed to high, non-physiological levels of cytokines to study OA provide simplified and focused analyses but fail to capture key signals from multi-cellular interactions (Johnson et al., 2016; Makarczyk et al., 2021). Thus, there is an impetus to design and develop 3D cell cultures that allow for more physiologically relevant interactions mediated by unidirectional and reciprocal cell communication, providing a better surrogate for early-stage PTOA (Samavedi et al., 2017; Peck et al., 2018; Hamasaki et al., 2021; Dwivedi et al., 2022). Most published models of early PTOA use *in vitro* tissue formats in which the interaction between two cell types is evaluated; they highlight the importance of cross-talk between cell types (Blasioli et al., 2014; Rothbauer et al., 2020; Hamasaki et al., 2021). Increasing evidence of the complex interplay between the synoviocytes and cartilage in the early inflammatory phase necessitates an *in vitro* model that can replicate cell-to-cell communication between the cell types involved in the early phase of PTOA (Griffin and Scanzello, 2019; Dilley et al., 2023). For example, an *ex vivo* model using human osteochondral plug–synovium explants was developed to study the role of mechanical injury and inflammation in the initiation of early PTOA (Dwivedi et al., 2022). Inclusion of the synovial component and a single injurious unconfined compression of the cartilage surface induced features relevant to PTOA-like initiation and progression; however, no significant differences were noted between the mechanically loaded *versus* control groups as the injury-induced microdamage preceded early inflammation (Dwivedi et al., 2022). Although the aforementioned model is a useful research tool for evaluating OA therapeutics, limitations of the study center on the availability of donor samples and tissues with no history of PTOA or OA and the inherent variability of the tissue response to mechanical injury.

The proposed study addresses this limitation by leveraging Fnf-induced inflammation to fill this gap and provide a valuable, flexible, and manipulable platform for investigating cellular behavior in both healthy and diseased states. Thus, the inclusion of macrophages and fibroblast-like synoviocytes (FLSs) is proposed, which can provide an endogenous source of cytokines upon stimulation with Fnfs rather than providing a predetermined cocktail of exogenous inflammatory molecules that is unlikely to reproduce synovial activation (Raychaudhuri and Raychaudhuri, 2010; Raychaudhuri et al., 2011; Takano et al., 2016; Bhattaram and Chandrasekharan, 2017; Bhattaram and Jones, 2019).

We hypothesize that an *in vitro* model incorporating synoviocytes that mediate joint inflammation, along with chondrocytes, can mimic the early stage of PTOA. Thus, a triculture model incorporating chondrocytes, synovial fibroblasts, and macrophages was established and exposed to Fnfs to activate inflammatory signaling cascades in all the cell types. Our temporally collected data on select biomarkers, cytokine profiles, and immunofluorescence analyses will demonstrate the ability of the triculture model to mimic the early stage of PTOA.

## 2 Materials and methods

### 2.1 Materials

THP-1 monocytic cells (ATCC, United States), human articular chondrocytes (HACs; Cell Applications, United States), and FLSs (Cell Applications, United States) were used. RPMI, DMEM/F12 and DMEM, fetal bovine serum (FBS), antibiotic–antimycotic solution, ascorbic acid, phorbol 12-myristate 13-acetate (PMA), lipopolysaccharide (LPS), and 4% paraformaldehyde (PFA) were obtained from Thermo Fisher Scientific (United States). Fibronectin (∼450 kDa; FC010) was purchased from Sigma-Aldrich (United States), and agarose-immobilized trypsin digestion kits were from ProteoChem (United States). Centrifugal concentrators (20 mm), Tris-glycine and Bis-Tris NuPAGE gels, a SilverQuest™ Silver Staining Kit, DAPI, ProLong™ Diamond Antifade Mountant, and rabbit anti-pNFκB p65 (MA5-15160) and anti-CD197 (MA5-31992) antibodies were purchased from Invitrogen (United States). Alexa Fluor 488-conjugated goat anti-rabbit IgG (ab150077) was obtained from Abcam (United Kingdom). IFN-γ and DuoSet ELISA kits (DY210, DY201, and DY279) were obtained from R&D Systems (United States). The Human Inflammation Array Q1 Kit was obtained from RayBiotech (United States). RNA extraction and qRT-PCR reagents, including the PureLink RNA Mini Kit (Thermo Fisher Scientific, United States) and the TaqMan RNA-to-CT 1-Step Kit (Life Technologies, United States), were used. Transwell tissue culture plates were from Corning (United States). MMPSense® 750 FAST substrate was purchased from PerkinElmer (United States). Imaging and quantification were performed using the Zeiss LSM 700 Confocal Microscope, the IVIS Lumina XR System, ImageJ™ software, and Living Image 4.0 software. GraphPad Prism (GraphPad, United States) was used for statistical analysis.

### 2.2 Cell culture

THP-1 monocytic cells were cultured in RPMI medium supplemented with 10% FBS and 1× antibiotic–antimycotic solution. THP-1 suspension cells were seeded in T-flasks at 10^5^ cells per mL and grown to 10^7^ cells per mL. Typically, THP-1 cells were transferred to 6-well plates and differentiated into the macrophage-like phenotype (M0), as detailed in Section 2.3.2. HACs and FLSs were cultured in DMEM/F12 and DMEM, respectively, and both were supplemented with 10% FBS and 1× antibiotic–antimycotic solution. For HACs, 25 μg/mL ascorbic acid was added to the media prior to use.

### 2.3 Preparation of Fnfs and their ability to activate M0 cells

#### 2.3.1 Fnf preparation and characterization

Intact fibronectin (∼450 kDa) was digested following the manufacturer’s protocol, which used agarose-immobilized trypsin in 0.1 M ammonium bicarbonate buffer (pH 8.0) at 37 °C for 8 h with end-to-end rotation. Following digestion, the supernatant containing Fnfs was centrifuged, concentrated using 20-mm filters, and analyzed by SDS-PAGE using 4%–12% Tris-glycine gels. A total of 2 µg protein was loaded per lane and run at 200 V for 50 min under reducing conditions. Protein bands were visualized using a SilverQuest™ Silver Staining Kit.

#### 2.3.2 Fnfs-induced macrophage differentiation

A total of 5 × 10^5^ THP-1 cells were seeded per well and differentiated into macrophage-like phenotype (M0) using 64 ng/mL PMA (100 nM) for 48 h (Chang et al., 2021; Hastings et al., 2023; Jo et al., 2024). M0 cells were rested in fresh RPMI media for two media changes (Daigneault et al., 2010). To evaluate the ability of Fnfs to activate M0, media containing 10 μg/mL of Fnfs were added to M0 cells for 72 h. LPS-treated M0 cells served as the positive control, where M0 cells were treated with 20 ng/mL IFN-γ (Genin et al., 2015; Baxter et al., 2020) for 12 h followed by a 24-hour-incubation with 20 ng/mL of LPS (Ishida et al., 2023).

### 2.4 Triculture Assembly

**FIGURE 1:**
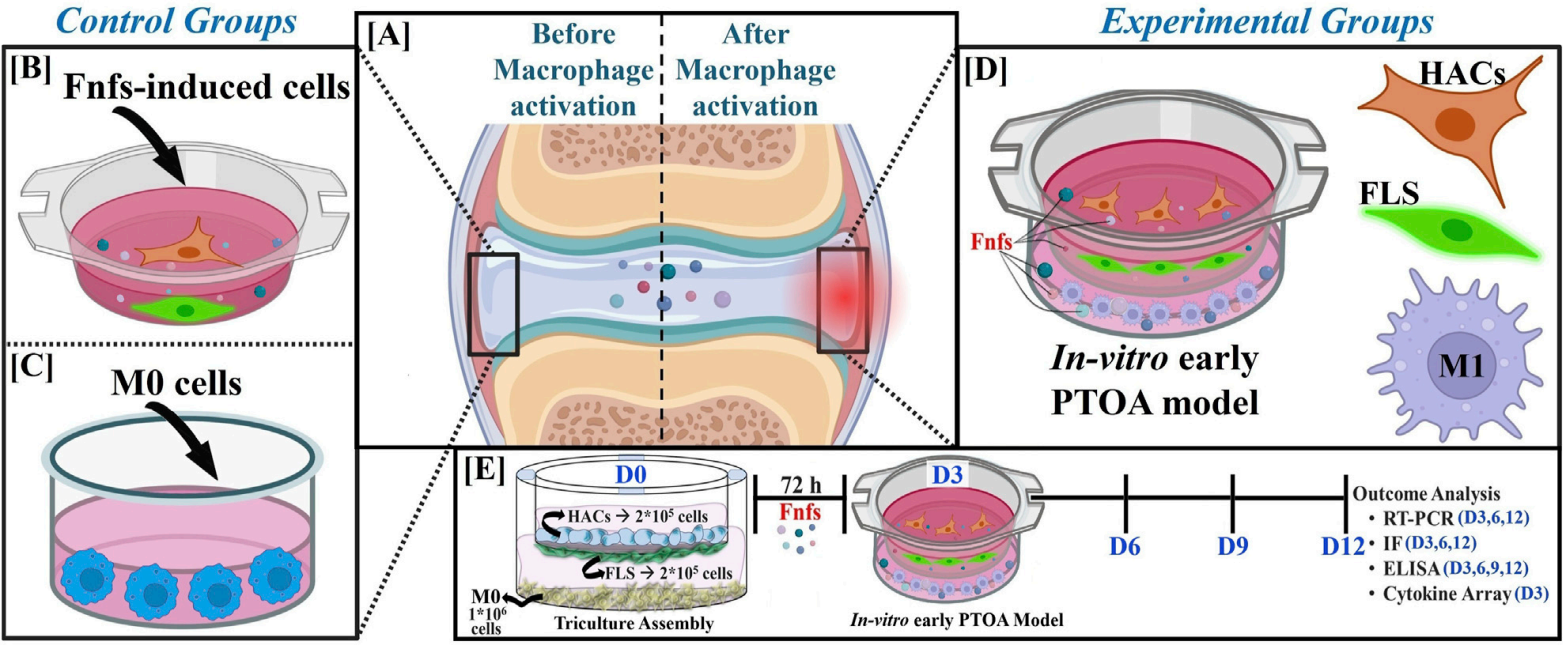
Design and experimental layout of the *in vitro* early post-traumatic osteoarthritis (PTOA) model. **(A)** Schematic of Fnf-induced early inflammation in selected cell types; **(B,C)** control groups; **(D)** experimental group: an *in vitro* early PTOA model was assembled, as described in Supplementary Figure S1, and treated with fibronectin fragments (Fnfs). **(E)** The triculture assembly was maintained in culture for 12 days. Samples were collected at indicated time-points and subjected to the outcome analyses listed.

Transwell tissue culture plates (TTCPs) housing a permeable polycarbonate membrane insert with a pore size of 0.4 µm were used to assemble the model (n = 3), and the procedure is schematically depicted in Supplementary Figure S1. Initial experiments were conducted to correlate RNA yield with cell numbers, which guided the cell numbers noted in Figure 1E. THP-1 cells were seeded at the bottom of the 6-well TTCPs and converted to M0 cells as per the method described earlier. Simultaneously, FLSs were seeded on the basal surface, and HACs were seeded on the apical surface of the transwell insert. The assembly was brought together, termed the triculture assembly (Supplementary Figure S1), and subsequently maintained in RPMI medium.

### 2.5 Establishment of the *in vitro early PTOA model*

**FIGURE 2:**
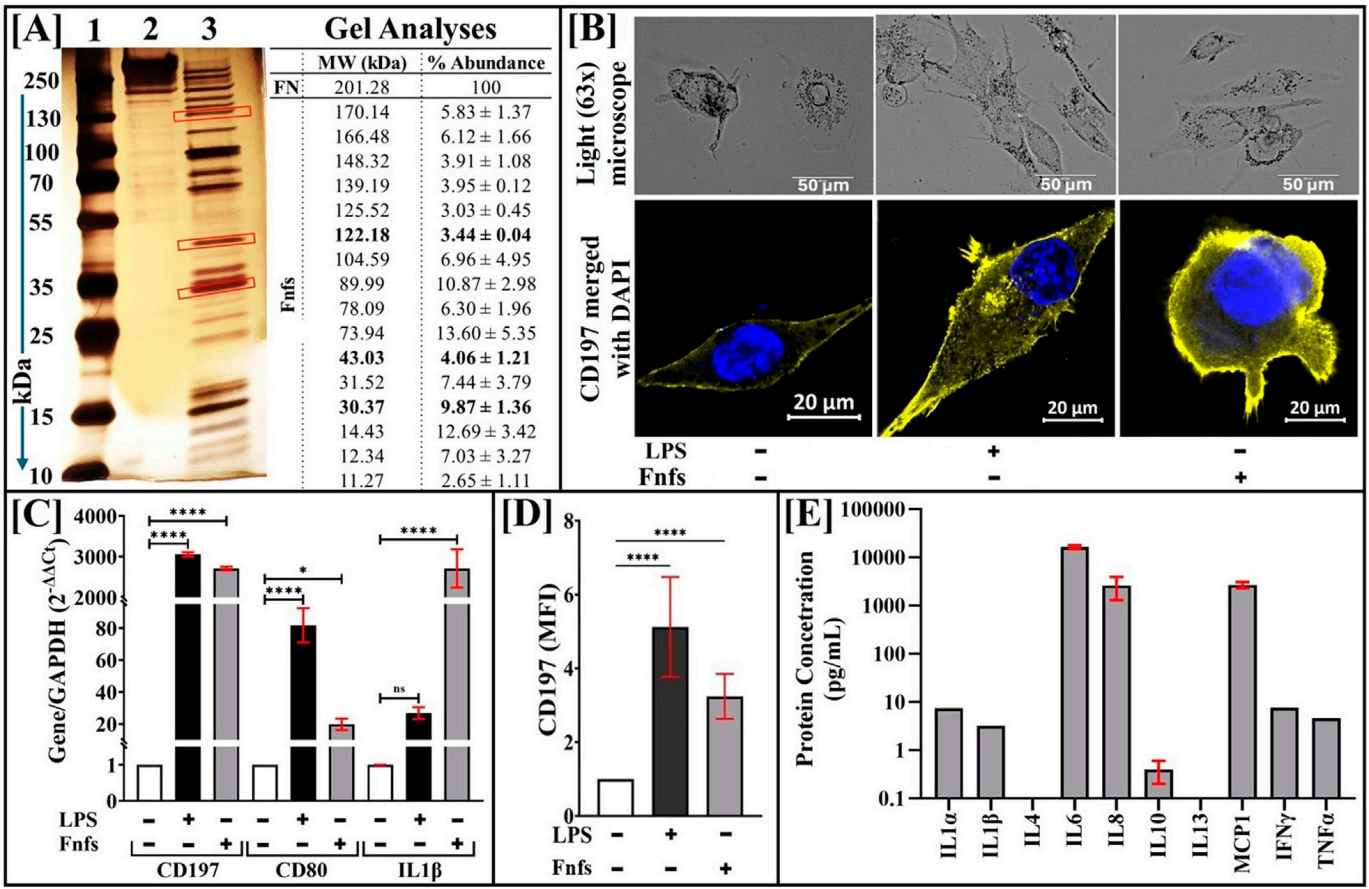
Preparation of Fnfs and their role in inducing the M1 macrophage phenotype. Fnfs were added to macrophage-like phenotype (M0) to generate Fnf-derived M1 macrophages, with LPS-treated M0 cells serving as a positive control. **(A)** Fnfs were prepared by digesting intact fibronectin with immobilized trypsin, separated on a 4%–20% Tris-glycine SDS-PAGE gel, and visualized by silver staining. Lane 1 shows the molecular weight ladder (10 kDa–250 kDa), lane 2 shows intact fibronectin, and lane 3 displays the resulting Fnfs. Protein bands were quantified using ImageJ™ to determine the relative abundance of key fragments. **(B)** To assess the phenotype, M0 cells (-Fnfs and -LPS) seeded on coverslips were treated with Fnfs or LPS and analyzed by light microscopy and immunofluorescence (IF) for CD197 expression. Scale bars are indicated in the figures. **(C)** In parallel, adherent cells were lysed for qRT-PCR to measure the gene expression of M1 markers. **(D)** IF signal intensity of panel B was quantified using ImageJ™. **(E)** Culture supernatants from Fnf-treated cells were collected and analyzed for cytokine levels using the Quantibody Cytokine Array™ (RayBiotech, GA).

Activation of rested M0 cells in triculture assembly was initiated with Fnfs (10 μg/mL in 3 mL) and termed the *in vitro* early PTOA model. Half-media changes were performed every 3 days for the remainder of the culture period. Cells and supernatants were harvested at specified time points for analysis as detailed in Figure 1E. The following formats were included as comparative controls: (1) HAC + FLS co-cultures without macrophages treated with Fnfs (Figure 1B) and (2) M0 macrophages cultured alone (Figure 1C). In separate experiments, M0 cells in the triculture assembly activated with IFN-γ + LPS served as the positive control and were termed LPS-induced triculture.

### 2.6 Immunofluorescence staining

For immunofluorescence (IF) staining, a coverslip with 25-mm diameter (n = 3) was placed at the bottom of TTCPs prior to seeding THP-1 cells to promote macrophage adherence. At the end of the experiment, these coverslips, along with the transwell membrane insert containing HACs + FLSs, were removed from TTCPs, washed with HBSS, and subjected to the IF staining process. In brief, cells were fixed with 4% PFA, blocked with 2% goat serum in TBST (TBS with 0.1% Tween-20), and incubated overnight at 4 °C with rabbit anti-phospho-NFκB p65 antibody (1:1,000) or rabbit anti-CD197 antibody (1:200). After washing, cells were incubated with Alexa Fluor 488-conjugated goat anti-rabbit IgG (1:1,000) for 1 h at room temperature. Nuclei were stained with DAPI using ProLong™ Diamond Antifade Mounting Media. Coverslips were directly mounted onto the glass slide, while membranes were mounted between a coverslip and the slide. Images were captured using a Zeiss LSM 700 Confocal Microscope at ×63 magnification. Fluorescent intensities were quantified using ImageJ™ software (n = 75–100 cells) by measuring the mean with respect to the area and integrated density.

### 2.7 ELISA and cytokine array

Supernatants were analyzed using a DuoSet ELISA Kit for MCP-1/CCL2, following the manufacturer’s instructions. Absorbance was measured at 450 nm with background correction at 540 nm. Samples were loaded in triplicate. The Human Inflammation Array Q1 Kit was used as per the manufacturer’s protocol and scanned using a fluorescent laser scanner at RayBiotech, GA.

### 2.8 RNA isolation and qRT-PCR

RNA was extracted using the PureLink RNA Mini Kit. Quantitative real-time PCR was performed using the TaqMan RNA-to-CT 1-Step Kit on a QuantStudio 3 System. Primers were selected as follows: CD80 (Hs01045161_m1) and CD197 (Hs01013469_m1) were used as the M1-specific markers due to their established roles in antigen presentation and inflammatory macrophage migration (Schulz et al., 2019; Nikonova et al., 2020; Mily et al., 2020). IL-1β (Hs01555410_m1) and TNF-α (Hs00174128_m1) were chosen as hallmark pro-inflammatory cytokines broadly upregulated in inflamed joint environments (Li et al., 2025). MMP3 (Hs00968305_m1), being a key mediator of synovial matrix breakdown, was selected based on its predominant expression in FLSs (Tolboom et al., 2002; Zeisel et al., 2005), and MMP13 (Hs00942584_m1), the principal collagenase in cartilage degradation, was selected for HACs (Hu and Ecker, 2021). pNFκB (Hs00765730_m1) was used as a shared upstream regulator of inflammatory gene expression (Giridharan and Srinivasan, 2018; Downton et al., 2023), and GAPDH (Hs02786624_g1) served as the housekeeping control. Samples were run in triplicate, and the relative expression was calculated using the 2^(-ΔΔCt) method.

### 2.9 *In vivo* intra-articular injection and IVIS imaging

All animal protocols and experimental procedures were approved by the Institutional Animal Care and Use Committee (IACUC #23-0481) of the University of Tennessee Health Science Center. Fnfs were sterile-filtered and injected into the left knee joints of anesthetized mice using 29-gauge needles through the patellar tendon. Anesthesia was induced and maintained with 2% isoflurane in oxygen (O_2_ is 300 mL/min) until non-responsive to toe pinch. After injection, joints were gently flexed and extended to distribute the material uniformly. Mice were monitored during recovery. For the *in vivo* imaging system (IVIS) imaging, MMPSense® 750 FAST substrate (100 μl) was retro-orbitally injected into sedated mice. After 24 h, animals were imaged using an IVIS Lumina XR System (Revvity Inc.). Fluorescence from the region of interest (ROI) was quantified using Living Image 4.0 software. Background fluorescence was subtracted, and the data were expressed as radiant efficiency. Post-imaging, mice were euthanized by carbon dioxide (CO_2_) asphyxiation in a chamber slowly filled at a displacement rate of 10%–30% of the chamber volume per minute. Following the cessation of respiration, a secondary method, cervical dislocation, was performed to ensure death in accordance with our IACUC-approved protocols, and the joints were harvested for qRT-PCR (Bedingfield et al., 2021; Bhatti et al., 2022).

### 2.10 Statistical analysis

Data are presented as the mean ± SD. An unpaired t-test with Welch’s correction was used to assess statistical significance. Significance was defined as follows: ns (not significant), *p < 0.05, **p < 0.01, ***p < 0.001, and ****p < 0.0001. Graphs were created using GraphPad Prism.

## 3 Results

### 3.1 Digestion of fibronectin with immobilized trypsin yields fibronectin fragments that activate M0 cells

Our experimental design utilizes Fnfs to initiate the activation of M0 cells in the *in vitro* model of early PTOA; first, intact fibronectin was digested with immobilized trypsin, and the resulting fragments were analyzed by SDS-PAGE and shown in Figure 2A. The apparent molecular weights of the protein bands in the digestate were determined by comparing their relative mobilities with the relative mobilities of standard proteins under the same conditions (Hedrick and Smith, 1968). Band intensities in the digestates were assessed by Image J™, and the percentage of relative abundance of the ∼30 kDa, ∼45 kDa, and ∼140 kDa bands in the digestate is 10.45% ± 0.9%, 10.37% ± 2.83%, and 3.50% ± 1.61%, respectively. Fibronectin fragments with molecular weights of 30, 45, and 140 kDa have been reported to trigger catabolic responses in cultured chondrocytes or cartilage explants (Peters et al., 2002).

The resulting adherent M0 cells obtained from the addition of PMA to THP-1 cells were assayed for the expression of pNκB and cytokine markers, and elevated levels compared to THP-1 were noted, alluding to priming (data not included). To assess the functionality of Fnfs, M0 cells were treated with Fnfs, and the resultant macrophages were assayed for M1 phenotype and markers, as shown in Figure 2. The light microscopy images shown in Figure 2B indicate changes in the cell morphology of M0 cells upon exposure to Fnfs. Immunofluorescence analysis confirmed the surface expression of CD197 on Fnf-treated M0 cells (Figure 2B/D), which is consistent with M1. Upon exposure to Fnfs, M0 cells exhibited increased expression of M1 markers, including CD80 and CD197, along with the pro-inflammatory cytokine IL-1β, as shown by qRT-PCR (Figure 2C). Furthermore, Fnf-derived macrophages secreted high levels of IL-6, IL-8, and MCP-1 into the media, as quantified by a cytokine array (RayBiotech, GA; Figure 2E). These findings indicate that Fnfs effectively drive the transition of M0 cells to M1 macrophages (Figure 1). LPS, a known M1 polarizing agent, was used as a positive control.

To model the early events in a post-traumatic joint environment, a triculture assembly was created by incorporating three main cell types, namely, articular chondrocytes, fibroblast-like synoviocytes, and macrophages. These cells were selected based on their central roles in initiating and sustaining inflammation and matrix degradation upon joint trauma. The initiation of the early inflammatory cascades in the triculture was attained with the addition of Fnfs, as shown in Figure 1. Activation of M0 cells in the triculture by Fnfs constitutes the main treatment group. LPS that potentiates chronic inflammation was included as a positive control. Transwell inserts containing both HACs and FLSs exposed to Fnfs served as appropriate controls. Samples from individual cells, while being a part of the triculture, were temporally collected, and the outcomes were assessed as indicated in Figure 1.

### 3.2 In the triculture PTOA model, Fnf-activated macrophages maintain the M1 phenotype

**FIGURE 3:**
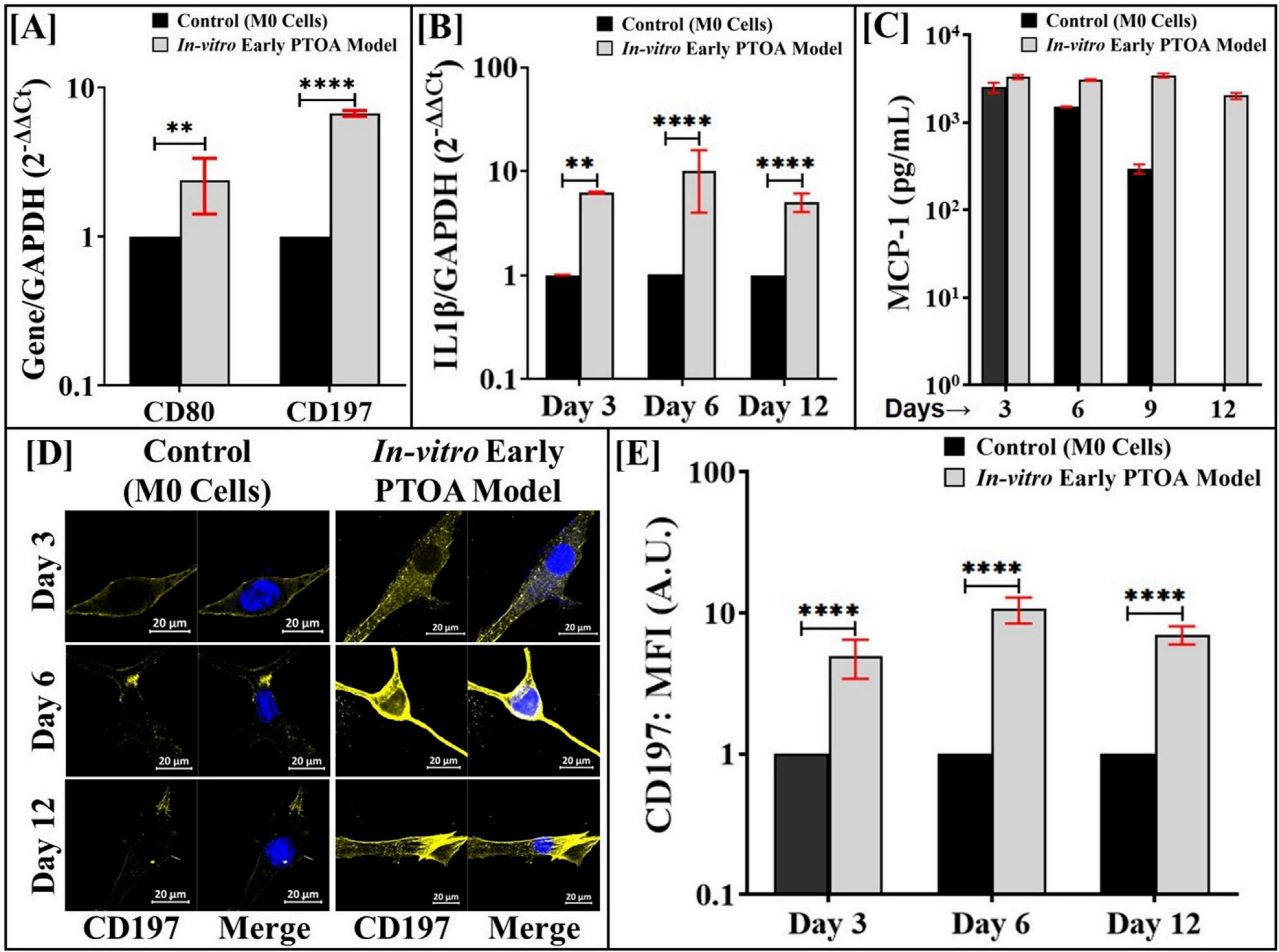
Fnf-derived macrophages maintain the M1 phenotype in the *in vitro* model of early PTOA. The M0 cells in the triculture assembly were activated with Fnfs, and the resulting *in vitro* model of early PTOA was maintained as shown in Figure 1. **(A)** Gene expression of M1 markers on day 3. **(Β)** Gene expression of IL1-β at indicated time points. **(C)** Protein expression of MCP-1 estimated by ELISA at indicated time points. **(D)** CD197 in M1 was visualized upon staining with antibodies and immunofluorescence imaging. **(E)** IF images were quantified using ImageJ™ (n = 75–100 cells). Parallel cultures of M0 cells (see Figure 1C) served as appropriate controls. Samples were analyzed in triplicates (n = 3); ns (not significant), *p < 0.05, **p < 0.01, ***p < 0.001, and ****p < 0.0001.

To ascertain the ability of the Fnfs to generate M1 macrophages in the triculture, the gene expression of M1 markers in the Fnf-activated macrophages on day 3 is shown in Figure 3A, where M1 macrophages showed increased expression of CD80 and CD197 compared to that in M0 cells. The elevated expression levels of the M1 markers indicated that Fnfs induced the transition to the M1 phenotype. Figures 3B,C show that macrophages in the triculture maintained the M1 phenotype, as evidenced by the elevated (∼10-fold) gene expression of IL-1β and protein expression of MCP-1, respectively. Figures 3D,E show the elevated level of CD197 expression in M1 cells in the triculture compared to that in control cultures that included M0 cells, indicating that the M1 phenotype was maintained throughout the culture. Similar analyses were performed with LPS and are shown in Supplementary Figure S4. Some differences were noted between the LPS- and Fnf-treated macrophages; for example, on day 3, a 10-fold higher expression of CD80 was noted in LPS-activated macrophages (Supplementary Figure S4A) compared to that in Fnf-activated macrophages (Figure 3A), indicating that LPS potentiates terminal differentiation while Fnfs bias differentiation toward the M1 phenotype. Upon Fnf treatment, IL-1β expression in macrophages remained elevated throughout the culture period (Figure 3B), whereas in macrophages treated with LPS, IL-1β was noted to reduce (Supplementary Figure S4B).

### 3.3 In the triculture PTOA model, Fnfs induce early inflammatory response in co-cultured HACs

**FIGURE 4:**
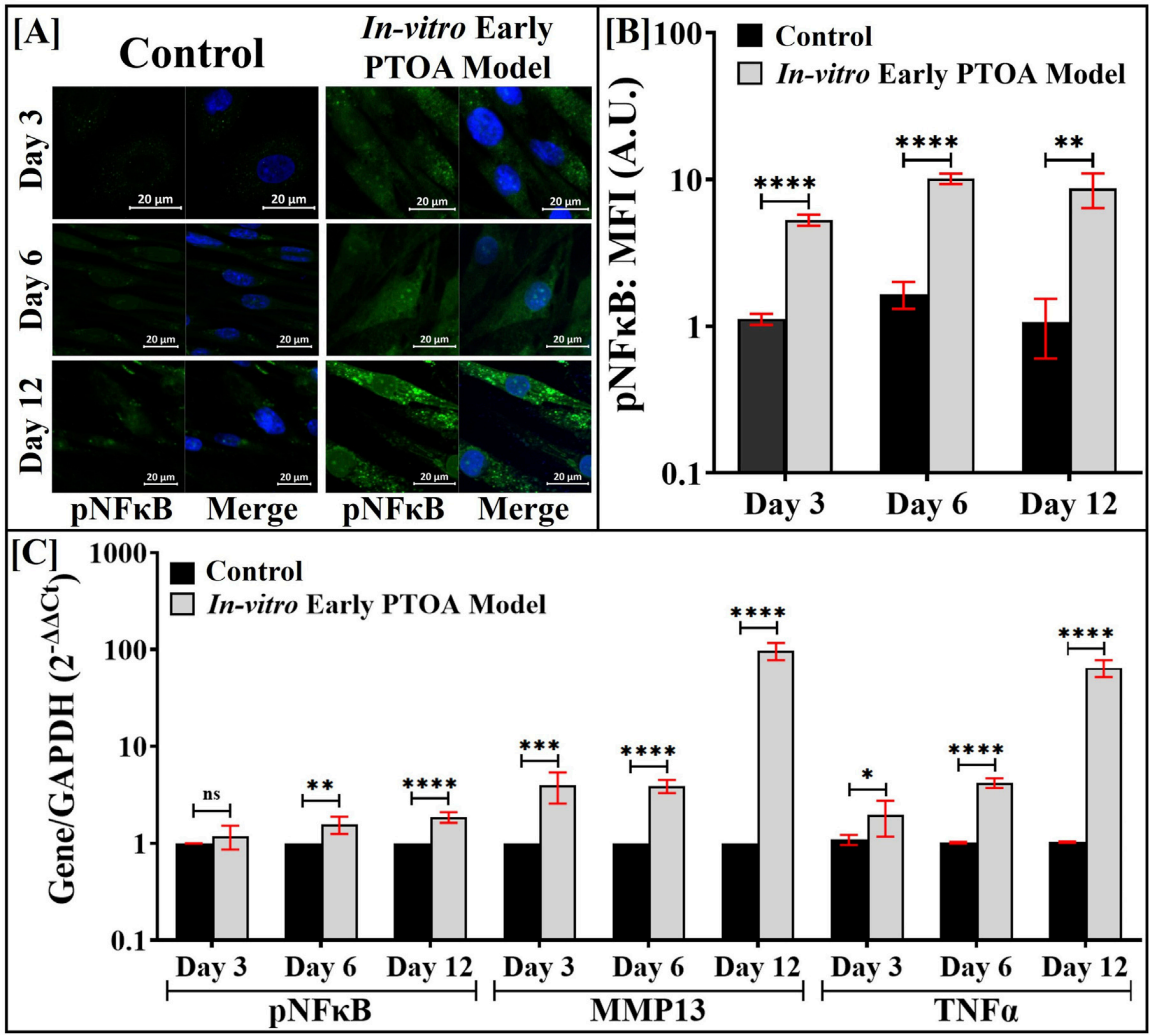
In the PTOA model, Fnf-derived M1 macrophages induce the expression of inflammatory markers in co-cultured HACs. The M0 cells in the triculture assembly were exposed to Fnfs, and the resulting *in vitro* model of early PTOA was maintained as shown in Figure 1. **(A)** pNFκB in HACs was visualized by IF imaging upon staining with anti-pNFκB antibodies. **(B)** IF images were quantified using ImageJ™ (n = 75–100 cells). **(C)** Expression of the indicated genes in HACs harvested from the apical surface of the triculture assembly by qRT-PCR. Parallel cultures of HACs co-cultured with FLSs (see Figure 1B) and exposed to Fnfs served as appropriate controls. Samples were analyzed in triplicate (n = 3); ns (not significant), *p < 0.05, **p < 0.01, ***p < 0.001, and ****p < 0.0001.

The localization of pNFκΒ in HACs was visualized by IF (Figure 4A), and the resultant nuclear localized fluorescent intensities were quantified and are shown in Figure 4B. During the first 3 days of culture, a 7-fold increase (*p* < 0.0001) in the nuclear-localized fluorescent intensity of pNFκB was noted in HACs that were maintained in the triculture compared to that in HACs in control cultures. During days 6 and 12, a ∼10-fold increase in the nuclear-localized fluorescent intensity of pNFκB was noted in HACs that were maintained in the triculture. Similar trends were noted with total pNFκB intensity (data not shown). Similar analyses were performed with LPS, and the nuclear-localized fluorescent intensity of pNFκB is shown in Supplementary Figure S2, where comparable levels were observed.

The gene expression of the selected inflammatory biomarkers was evaluated by qRT-PCR and is shown in Figure 4C. Compared to the control, on days 6 and 12, a significant increase in gene expression of pNFκΒ was noted in HACs. Elevated gene expression of MMP13 and TNF-α was also noted in HACs that were maintained in the triculture, with expression levels increasing (from 10- to 100-fold) over the culture period. Similar analyses were performed with LPS, and the gene expression of selected markers is shown in Supplementary Figure S2. Compared to that with Fnf treatment, expressions of MMP13 and TNF-α were observed to be significantly lower in LPS-treated macrophages.

### 3.4 In the *in vitro* model of early PTOA, Fnfs induce early inflammatory responses in co-cultured FLSs

**FIGURE 5:**
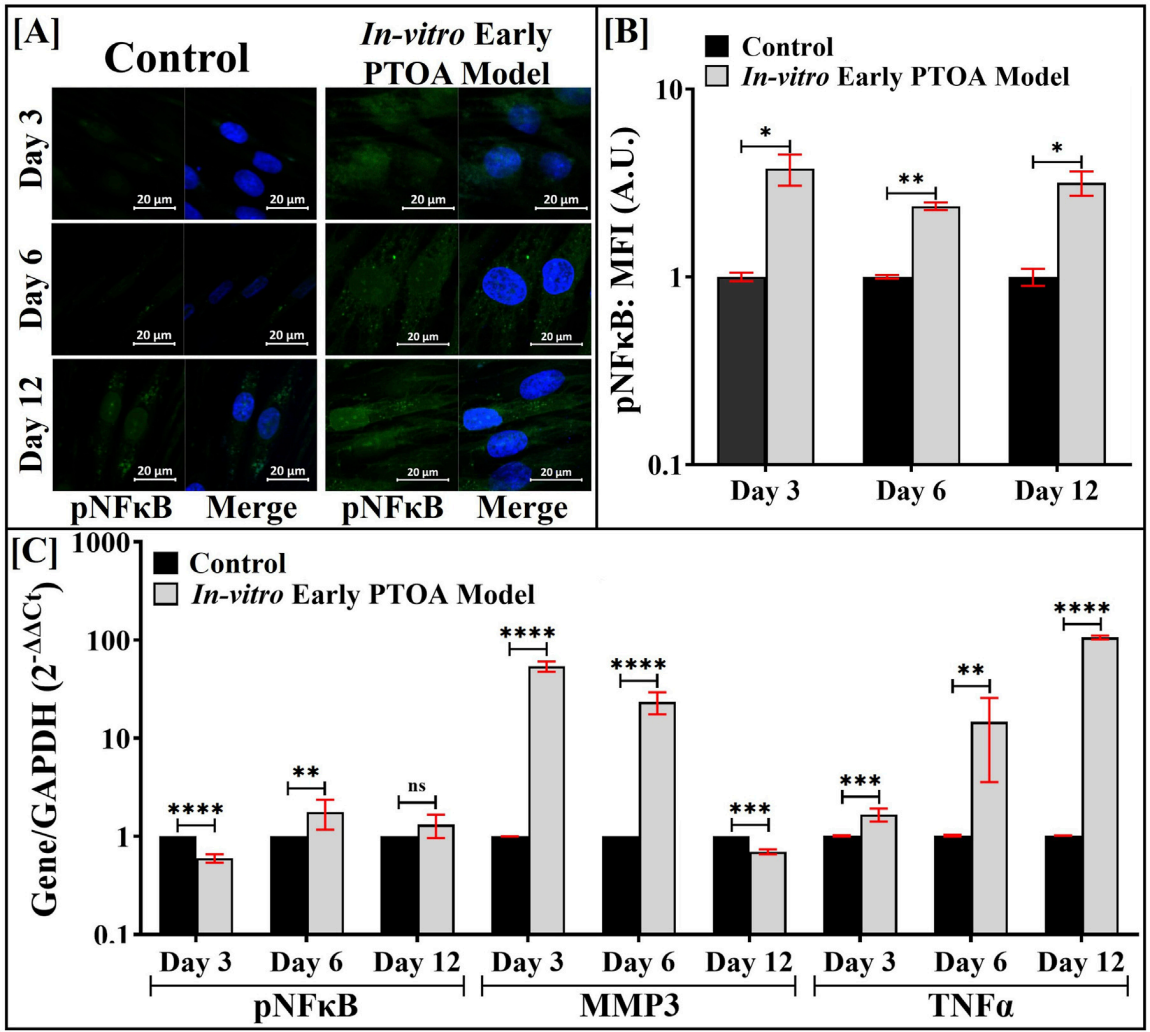
In the PTOA model, Fnf-derived M1 macrophages induce the expression of inflammatory markers in co-cultured FLSs. The M0 cells in the triculture assembly were exposed to Fnfs, and the resulting *in vitro* model of early PTOA was maintained as shown in Figure 1. **(A)** pNFκB in FLSs was visualized by IF imaging upon staining with anti-pNFκB antibodies. **(B)** IF images were quantified using ImageJ™ (n = 75–100 cells). **(C)** Expression of the indicated genes in FLSs harvested from the basal surface of the triculture assembly was quantified by qRT-PCR. Parallel cultures of FLS co-cultured with HACs and exposed to Fnfs (see Figure 1B) served as appropriate controls. Samples were analyzed in triplicate (n = 3); ns (not significant), *p < 0.05, **p < 0.01, ***p < 0.001, and ****p < 0.0001.

The localization of pNFκΒ in FLSs was visualized by IF (Figure 5A), and the nuclear-localized fluorescent intensity was quantified and is presented in Figure 5B. During the first 3 days of culture, an 8-fold (*p* < 0.001) increase in the nuclear-localized fluorescent intensity of pNFκB was noted in FLSs that were maintained in the triculture compared to that in control cultures. The level of pNFκB expression remained unaltered between days 6 and 12. Similar analyses were performed with LPS, and the corresponding nuclear-localized fluorescent intensity of pNFκB is shown in Supplementary Figure S3. The protein expression of pNFκB was noted to be similar.

The gene expression of selected inflammatory biomarkers was evaluated by qRT-PCR and is shown in Figure 5C. On day 6, a modest increase in the gene expression of pNFκB was observed in FLSs in the early PTOA model assembly compared to that in control cultures, with no significant differences on days 3 or 12. Elevated gene expressions of MMP3 and TNF-α were also noted in FLSs that were maintained in the early PTOA model assembly; however, on day 12, MMP3 showed a decrease. Similar analyses were performed with LPS, and the gene expression of the selected markers is shown in Supplementary Figure S3. Compared to macrophages treated with Fnfs, expressions of MMP3 and TNF-α were observed to be lower in LPS-treated macrophages.

### 3.5 Intra-articular injection of Fnfs enhances the expression of MMP3 and downregulates collagen-II expression

**FIGURE 6:**
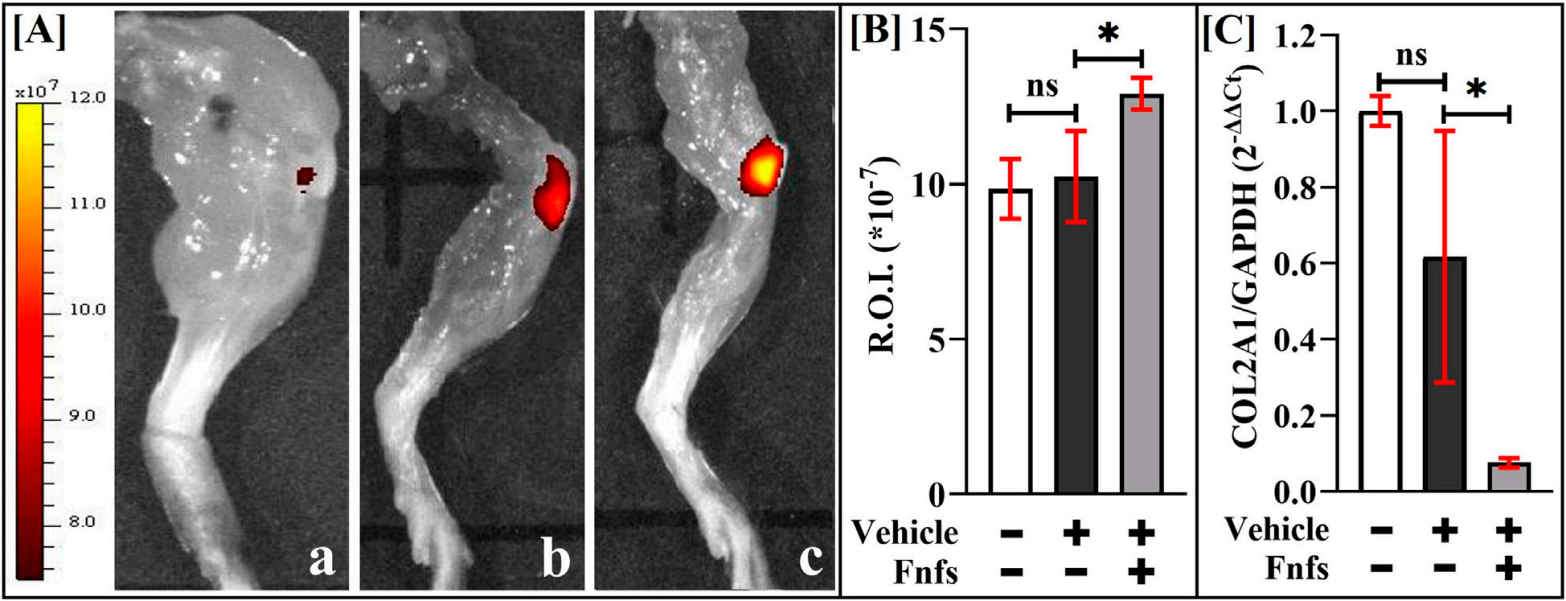
Intra-articular injection of Fnfs causes enhanced MMP fluorescence *in vivo* and downregulates the gene expression of *COL2A1*. 24 h-post Fnfs or diluent injection, mice were injected with MMPSense 750 and imaged using an *in vivo* IVIS system after 24 h. **(A)** IVIS images of knee joints, (a) control mice, (b) control mice injected with diluent, and (c) mice intra-articularly injected with Fnfs. **(B)** Fluorescence intensities were quantified within the region of interest (R.O.I) using Living Image 4.0 software. **(C)** Gene expression of COL2A1 at 48-h after Fnf injection. Samples were analyzed in triplicate (n = 3); ns (not significant), *p < 0.05, **p < 0.01, ***p < 0.001, and ****p < 0.0001.

To allow a direct comparison of the ability of Fnfs to initiate early inflammation, Fnfs were injected intra-articularly (I.A.) in mice. After 48 h of the injection of Fnfs, MMPSense was visualized using IVIS and is shown in Figure 6. Our data in Figure 6B show that injected Fnfs upregulated MMPs compared to diluent-injected control. Fnf injection significantly downregulated the gene expression of type-II collagen in micro-dissected joint cartilage (Figure 6C).

## 4 Discussion

Unresolved reparative processes upon traumatic injury lead to PTOA, a joint disease characterized by cartilage degradation and synovial inflammation (Dilley et al., 2023). Upon traumatic impact, proteolysis or fragmentation of the components of the ECM has been reported to regulate localized innate immune responses, which result from the recognition of specific molecules by pattern recognition receptors (PRRs) present in both immune and non-immune cells of the joint complex (Haseeb and Haqqi, 2013; Griffin and Scanzello, 2019). PRRs recognize two classes of specific molecules resulting either from the presence of pathogens, via pathogen-associated molecular patterns (PAMPs), or from damage-associated molecular patterns (DAMPs), which are released from damaged tissue or degraded ECM components. While PAMPs are external agents derived from viruses or bacteria (i.e., LPS), DAMPs represent endogenous stimuli, which are released either from proteolytically digested ECM fragments or from intracellular components (Dilley et al., 2023). The catabolic ECM components discovered to be capable of acting as DAMPs are glycoproteins, proteoglycans, glycosaminoglycans, and the soluble form of matrilin-2, which act as autonomous triggers of the inflammatory process through direct interaction with the specific PRRs via the respective TLRs (Frevert et al., 2018). Among the ECM fragments that can act as triggers of early inflammation, the best example and most characterized system is that of fragments of the matrix protein, fibronectin (Fn), Fnfs (Xie et al., 1992; Homandberg, 1999; Homandberg, 2001).

Fnfs produced during damage to the cartilage ECM have the potential to enhance early OA-like processes (Frevert et al., 2018). For example, a large fraction of fragmented fibronectin was observed in the synovial fluid of patients with chronic arthritis (Griffiths et al., 1989). Abundant Fnfs ranging from 30 kDa to 200 kDa were detected in the synovial fluids from patients diagnosed with OA or joint trauma (Griffiths et al., 1989). The Fnf concentrations ranged between 1.5 and 3.0 mM (Xie et al., 1992). While there are other fragment systems, Fnfs have been shown to enhance the levels of catabolic cytokines, as in OA, and upon intraarticular injection, they also caused nearly complete PG depletion in just 2 days (Homandberg et al., 2001). Thus, exogenously administered Fnfs have the potential to offer a model in which physiological levels of Fnfs can be used to examine aspects of tissue damage and repair induced by trauma, as opposed to exposing cultures to non-physiological levels of cytokines. Triculture assemblies that were exposed to Fnfs were noted to yield higher levels of inflammatory markers in the cell types studied compared to triculture assemblies where the M0 cells were not activated via Fnfs, suggesting the role of Fnfs in the generation of the early PTOA model (Supplementary Figure S5).

Temporal control of the early immune response is critical and necessitates a switch from pro-inflammatory to mediators required for the resolution of inflammation. Fnfs not only enhance cartilage damage but also cause an anabolic reparative response, thus allowing us to study the mechanism(s) by which damage and repair are coupled (Homandberg and Hui, 1994; Homandberg et al., 2001). In contrast, LPS potentiates robust, chronic inflammation (Liu et al., 2018; Ishida et al., 2023). In addition, Fnfs represent DAMPs that are released from the ECM and activate macrophages by engaging TLR2 and TLR4 receptors, which is similar to macrophage activation post-trauma. In contrast, activation of macrophages via LPS, an endotoxin, represents PAMPs and potentiates chronic inflammation. Understanding the mechanism by which Fnfs elicit this destructive process should aid in designing novel therapeutic approaches. Therefore, we sought to establish an *in vitro* model to temporally examine the cross-talk between chondrocytes, synovial fibroblasts, and Fnf-stimulated macrophages.

To the best of our knowledge, this is the first *in vitro* model combining the co-culture of human chondrocytes with the components of the synovium, namely, fibroblast-like synoviocytes and macrophages, and incorporating a physiological DAMP to study the initiation of inflammatory cascades in early PTOA, mimicking the events upon traumatic injury. Additionally, in contrast to using fibronectin fragments of a singular molecular weight, this study utilized a cocktail of Fnfs with reported catabolic activities (Figure 2). Furthermore, the levels of Fnfs used were comparable to levels noted in injured joints, thus mimicking the inflammatory stage observed in the early stage of PTOA (Homandberg et al., 1992). The developed early-stage *in vitro* PTOA model captures the complex interplay between macrophages, synovial fibroblasts, and cartilage in a pro-inflammatory milieu and provides a temporal snapshot of the inflammatory biomarkers in the impacted cell types. Compared to culture systems that use a singular cell type exposed to non-physiological levels of cytokines, our model allowed the evaluation of the contribution of Fnf-activated macrophages in inducing inflammation in synovial fibroblasts and normal chondrocytes.

The increase in MMP3 and MMP13 expression in FLSs and HACs, respectively, upon induction of the *in vitro* model of early PTOA by Fnfs validated the presence of catabolic mediators and upregulation in the production of cartilage-damaging proteinases, as reported elsewhere (Xie et al., 1992). The M1 phenotype was confirmed by the increase in pro-inflammatory cytokine secretion and gene expression, along with the expression of cluster of differentiation surface markers such as CD80 and CD197. The *in situ* macrophage phenotype induced by Fnfs also impacted the expression of the pro-inflammatory marker NFκB in HACs and FLSs, indicating the engagement of pro-inflammatory mechanisms in macrophages by Fnfs via TLR receptors (Hwang et al., 2015; Fei et al., 2018; Frevert et al., 2018). In the context of early PTOA, it is postulated that DAMP-activated synovial macrophages produce pro-inflammatory cytokines that activate the inflammatory cascades in FLSs and HACs (Griffin and Scanzello, 2019; Haseeb and Haqqi, 2013). We posit that the cascade was successfully replicated in the model used in this study, as evidenced by Figures 3–6. Exposure of Fnfs to HACs and FLSs in the absence of macrophages did not show appreciable levels of either pNFκB or gene expression of catabolic markers, suggesting the critical role of activated macrophages in mounting the inflammatory response.

**FIGURE 7:**
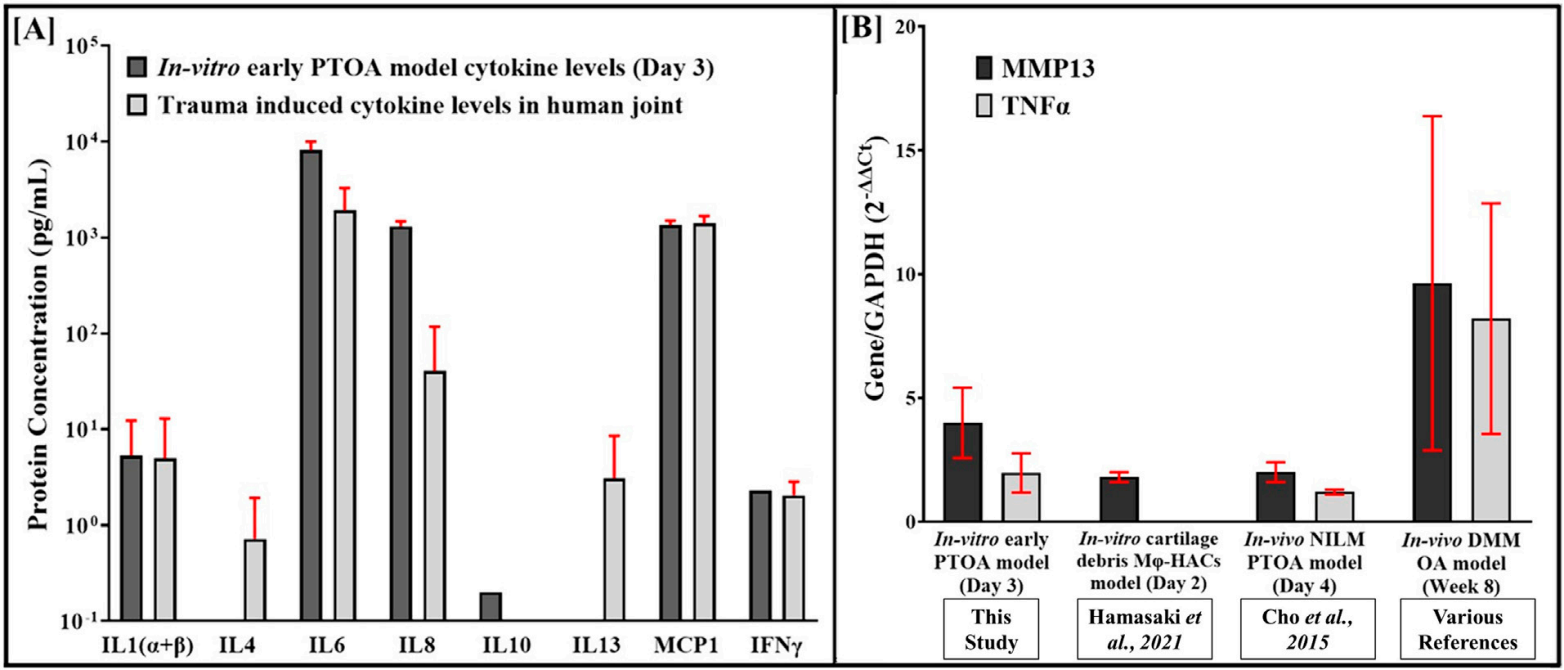
Comparison of the early inflammatory markers in the PTOA model with literature. **(A)** Media from the PTOA model on day 3 was analyzed using the Quantibody™ cytokine array (RayBiotech, GA), and the cytokine levels are reported (n = 3). The levels of the selected cytokines in the synovial fluid of healthy patients undergoing surgery were extracted from the literature and reported (Tsuchida et al., 2014). **(B)** Comparison of the gene expression of MMP13 and TNFα on day 3 between the PTOA model (this study; n = 3) with the reported *in vitro* and *in vivo* NILM early PTOA model (Cho et al., 2015) and *in vivo* DMM OA models (Leong et al., 2014; Qu et al., 2018; Cai et al., 2019; Lin et al., 2020).

In comparison, the observed expression of MMP13 at day 2 in the cartilage-debris-induced *in vitro* model reported elsewhere (Hamasaki et al., 2021) is more representative of macrophage activation due to the phagocytosis of cartilage fragments than the Fnf-induced activation of macrophages (Figure 7A). While the osteochondral-synovium co-culture *ex vivo* model developed elsewhere to study the earliest stages of PTOA progression upon injury (Dwivedi et al., 2022) shows distinct cellular, inflammatory, and matrix-related alterations relevant to PTOA-like initiation and progression, matrix breakdown upon a single mechanical impact injury preceded early inflammatory processes.

On day 3 of culture, the gene expression of MMP13 and TNF-α in HACs that were part of the triculture model was quantified by qRT-PCR and compared with the gene expression noted in micro-dissected cartilages on day 4 from mouse knee joints that were non-invasively mechanically loaded (NIML: *in vivo* early PTOA model) (Cho et al., 2015) and are shown in Figure 7A, and similar expression levels were noted. In contrast, higher levels of MMP13 and TNF-α were observed at week 8 in a late-stage destabilized medial meniscus (DMM)-OA model (Leong et al., 2014; Qu et al., 2018; Cai et al., 2019; Lin et al., 2020). The Quantibody Cytokine™ array was used to estimate the levels of select chemo- or cytokines in the media, as shown in Figure 7B, and to compare them with levels reported in the synovial fluid of joints with symptomatic cartilage defects (Tsuchida et al., 2014). Levels of pro-inflammatory cytokines (IL-1β, IFN-γ, MCP-1, and IL-6) were noted to be similar between the *in vitro* early PTOA model and those observed in early-phase *in vivo* joint models with focal defects or injuries (Tsuchida et al., 2014). Expression of anti-inflammatory cytokine IL-10 was noted in the *in vitro* early PTOA model. Upon traumatic injury, during the early inflammatory phase, a plethora of cytokines are noted *in vivo*; our analyses suggest that the *in vitro* early PTOA model proposed in this study holds promise for evaluating this phase. Our cumulative data demonstrate our ability to design and develop a triculture model for understanding the inflammatory response in early PTOA when exposed to Fnfs.

In contrast to end-point analyses that use histology, non-invasive techniques using fluorescent monoclonal antibodies that target damaged cartilage *in vivo*, in combination with dual fluorescence optical imaging, were used to detect the ability of intra-articularly injected Fnfs to induce early inflammation in a mouse model. A monoclonal antibody that does not activate the complement upon binding and causes no discernible histopathological changes in the host was used. Correlating with an increase in the MMP levels at the end of 48-h, injection of Fnfs led to a 10-fold decrease in the gene expression of COL2A1 in joint cartilage tissue. MMP-mediated collagenolysis has long been implicated (Amar et al., 2017). A similar observation regarding elevated MMP-3 levels in cartilage was noted when rabbit knee joints were injected with Fnfs (a mixture of 29 kDa and 50 kDa) (Homandberg et al., 2001). While a detailed study is needed, our cumulative data demonstrate that the Fnf-induced early PTOA model captures the early response feature of MMP expression noted in *in vivo* models.

While the use of procurable human cell lines, their culture for up to 14 days, and in the quantities required to permit detailed analysis is facile; future approaches will include procuring blood samples and human tissues, isolating cells, phenotyping them, and using them in models. To demonstrate the ability of Fnfs to initiate early PTOA in an *ex vivo* model, future experiments will use formats detailed elsewhere that use intact explant tissues from healthy human donors of both sexes. If successful, they will serve as a surrogate for early-stage PTOA and allow researchers to replicate the etiology of early PTOA, thus bridging the gap between animal and human systems.

## Data Availability

The raw data supporting the conclusions of this article will be made available by the authors, without undue reservation.
