# Supplementary material for: Synoviocyte–chondrocyte triculture model for early-stage PTOA: fibronectin fragment-induced catabolic effects in vitro and in vivo
Source: Front Bioeng Biotechnol. 2025 Dec 4;13:1683333. doi: 10.3389/fbioe.2025.1683333 (PMC12711863; doi:10.3389/fbioe.2025.1683333)
Supplement: Supplementary file 1 [file Supplementaryfile1.docx]

**Supplementary Figures**:


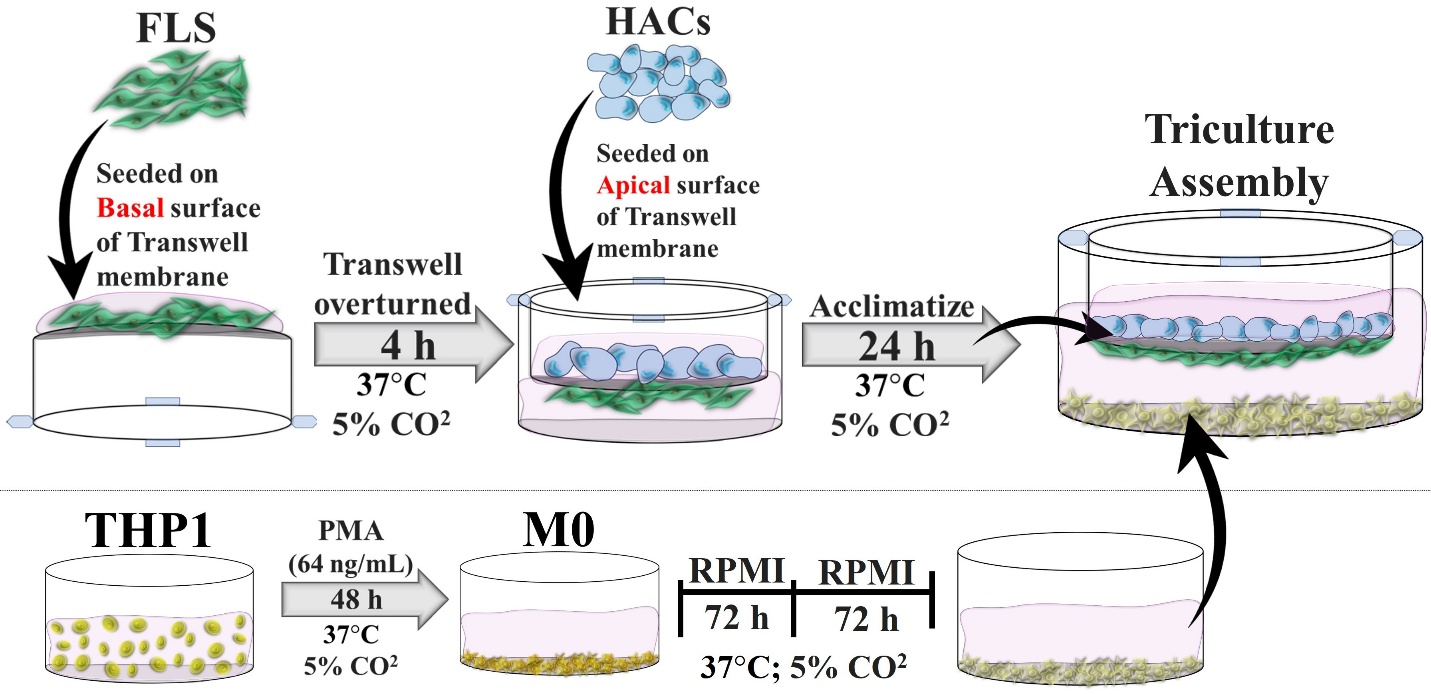
**Figure 1S: Schematic of the Triculture assembly**. A 6-well transwell format was employed, where human synovial fibroblasts (FLS) were seeded on the basal surface of the transwell-insert and allowed to adhere to the “polycarbonate” membrane for 4-hours. Subsequently, human articular chondrocytes (HACs) were seeded onto the apical surface and allowed to adhere for 24 hours. In parallel, a suspension of THP-1 cells was added to a 6-well TCP plate and converted to a macrophage-like phenotype (M0) using phorbol-12-myristate-13-acetate (PMA). The transwell insert housing both FLS and HACs were then brought into contact with the 6-well TCP plate containing M0 cells. The entire setup upon assembly was termed as “Triculture assembly”.


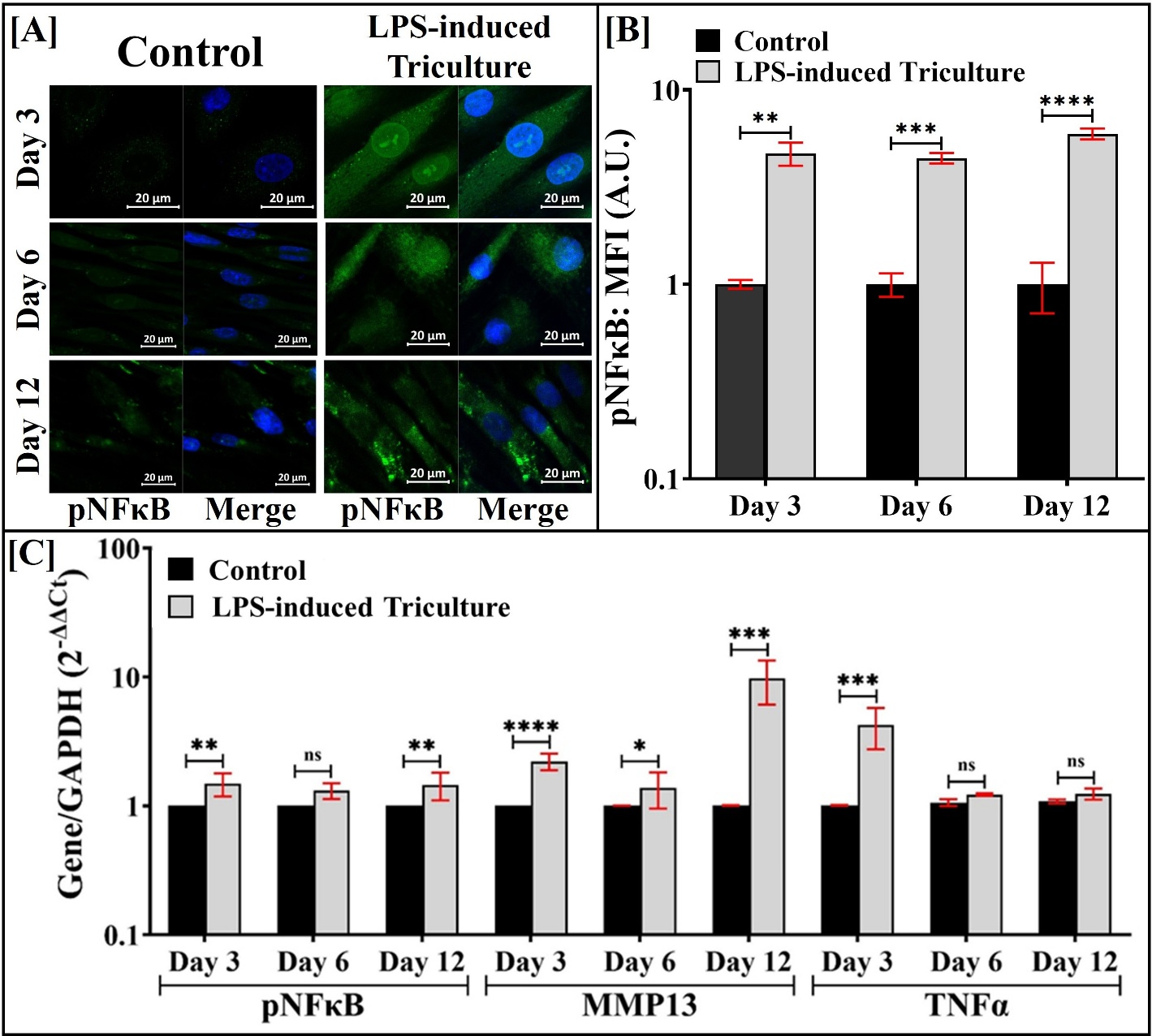


**Figure 2S: In the LPS-induced triculture, LPS derived M1 macrophages induce the expression of inflammatory markers in co-cultured HACs.** The M0 cells in the triculture assembly shown in figure 1S were exposed to LPS and cultured for 12 days. [A] pNFκb in HACs was visualized by IF imaging upon staining with anti-pNFκb antibodies; [B] IF images were quantified using Image J^TM^ (n=75-100 cells) and [C] Expression of indicated genes in HACs harvested from the basal surface of the triculture assembly were quantified by qRT-PCR. Parallel cultures of HACs co-cultured with FLS and exposed to LPS served as appropriate controls. Samples were analyzed in triplicates (n=3); ns (not significant), * p < 0.05, ** p<0.01, *** p<0.001, **** p < 0.0001.

**
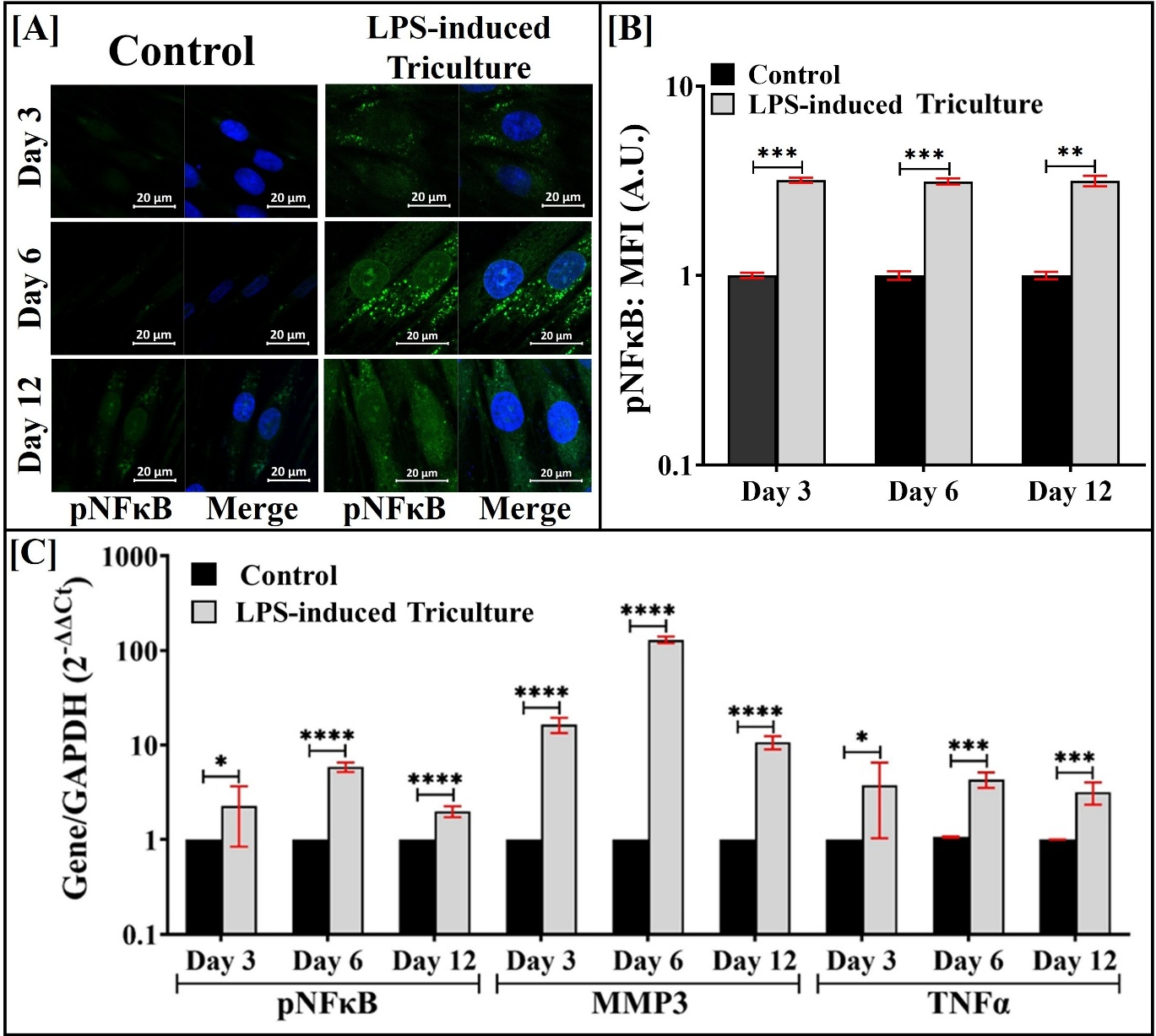
**

**Figure 3S: In the LPS-induced triculture, LPS derived M1 macrophages induce the expression of inflammatory markers in co-cultured FLS.** The M0 cells in the triculture assembly shown in Fig. 1S were exposed to LPS and cultured for 12 days. [A] pNFκβ in FLS was visualized by IF imaging upon staining with anti-pNFκb antibodies; [B] IF images were quantified using Image J^TM^ (n=75-100 cells) and [C] Expression of indicated genes in FLS harvested from the basal surface of the tri-culture assembly were quantified by qRT-PCR. Parallel cultures of FLS co-cultured with HACs and exposed to LPS served as appropriate controls. Samples were analyzed in triplicates (n=3); ns (not significant), * p < 0.05, ** p<0.01, *** p<0.001, **** p < 0.0001.


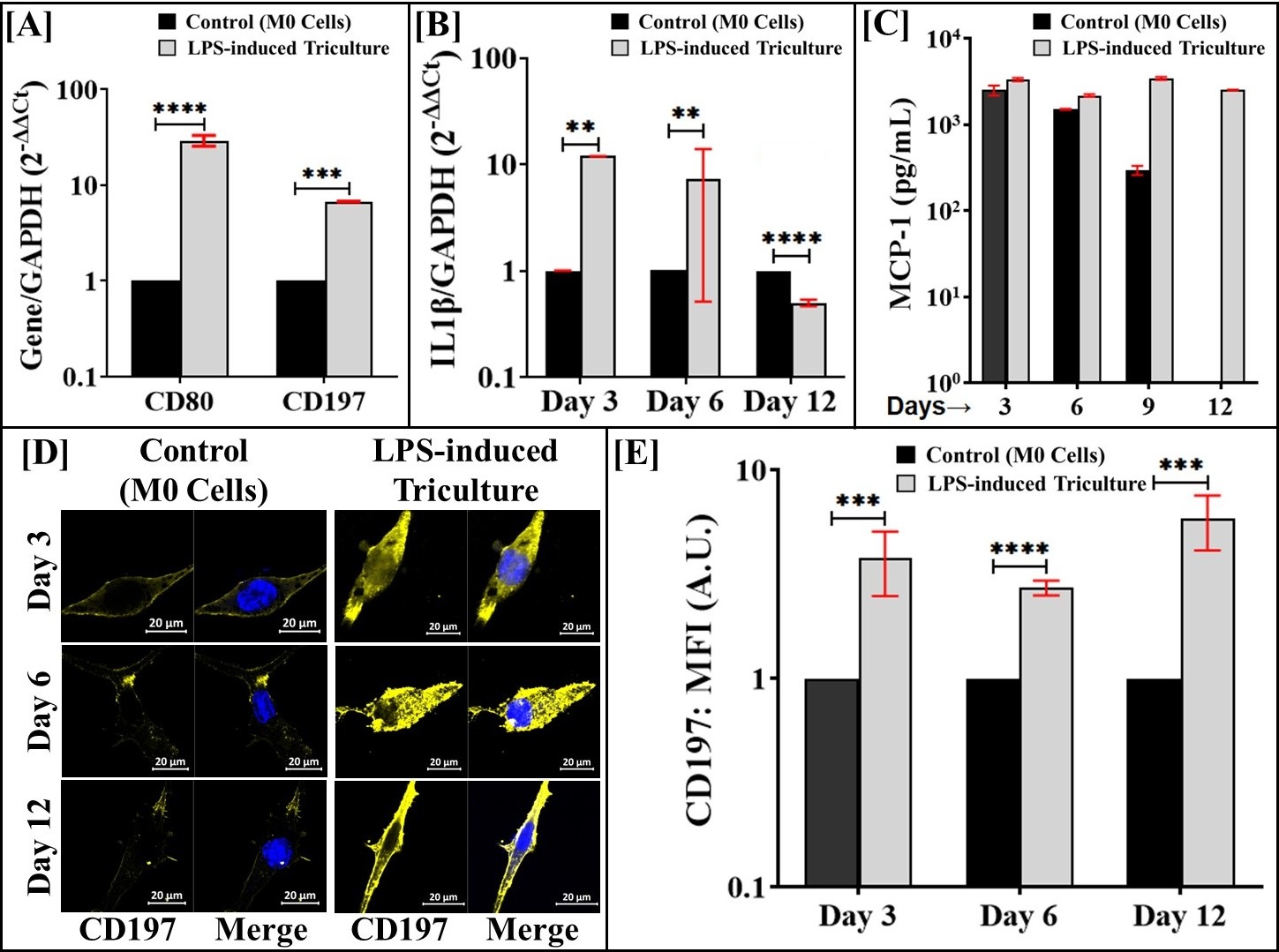
 **Figure 4S: In the LPS-induced triculture, LPS derived macrophages maintain the M1 phenotype.** The M0 cells in the triculture assembly shown in Fig. 1S were activated with LPS and cultured for 12 days. Adherent LPS-derived M1 macrophages from the TCP-plate were quantified by qRT-PCR: [A] Gene expression of M1 markers at day 3; [B] Gene expression of IL1β at indicated time points. [C] MCP-1 levels in the media supernatant were estimated by ELISA. [D] CD197 in M1 was visualized upon staining with antibodies and immunofluorescence imaging; [E] IF images were quantified by ImageJ^TM^ (n=75-100 cells). Parallel cultures of M0 cells served as appropriate controls. Samples were analyzed in triplicates (n=3); ns (not significant), * p < 0.05, ** p<0.01, *** p<0.001, **** p < 0.0001.

**
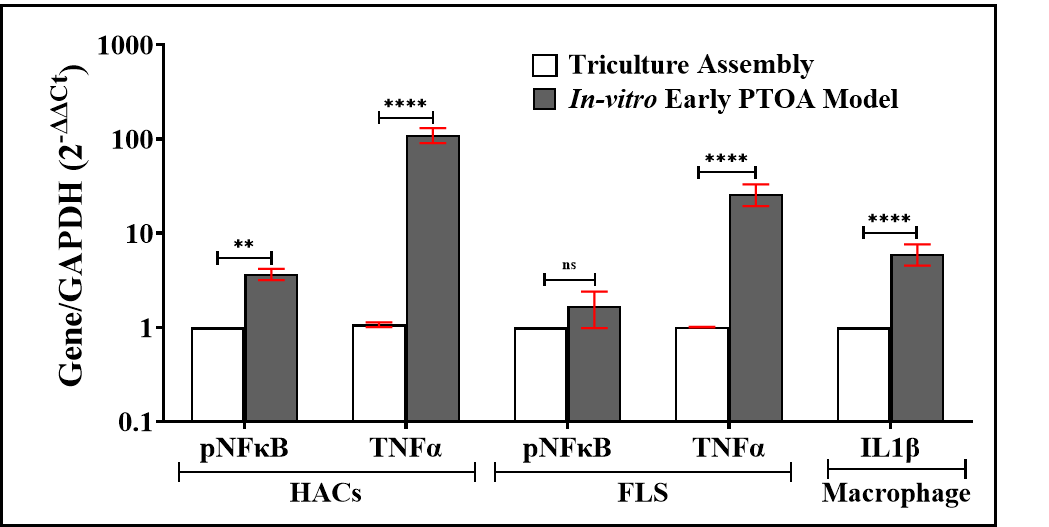
 Figure 5S: Gene expression of select markers in HACs, FLS and macrophages in the early PTOA model as compared to triculture assembly.** Both triculture assembly (Fig. 1S) and the early in vitro PTOA model (Fig. 1D) were cultured independently. HACs, FLS, and macrophages isolated from transwell tissue culture plates (TTCPs) were analyzed by qRT-PCR on day 12 for the indicated genes. Samples were analyzed in triplicates (n=3); ns (not significant), * p < 0.05, ** p<0.01, *** p<0.001, **** p < 0.0001.
